# Frequency of Thyroid Disorder in Pre- and Postmenopausal Women and Its Association With Menopausal Symptoms

**DOI:** 10.7759/cureus.40900

**Published:** 2023-06-24

**Authors:** Manjusha Yadav, Varsha Kose, Anuja Bhalerao

**Affiliations:** 1 Department of Obstetrics and Gynaecology, NKP Salve Institute of Medical Sciences and Research Centre, Nagpur, IND

**Keywords:** hyperthyroidism, hypothyroidisim, menopausal symptoms, post-menopausal women, pre-menopausal women, thyroid disorders

## Abstract

Background

Thyroid hormone has a significant effect on women’s life. Its dysfunction is widespread, particularly among women over 50. Understanding how thyroid disease's clinical symptoms change with aging is crucial when treating peri-and postmenopausal women. Considering that 30% of India's population is female, the thyroid disease epidemic appears to affect Indian women more than any other ailment. Therefore, the main aim of the study was the detection of the frequency of thyroid disorder in pre and postmenopausal women and its association with menopausal symptoms.

Methodology

An analytical cross-sectional study was conducted at a tertiary care centre. All premenopausal and postmenopausal women attending the obstetrics and gynaecology department above 40 years of age, achieving natural menopause, and willing to participate were included in the study.

Results

A total of 150 women above 40 years of age were included in the study out of which 61 females were premenopausal age and 89 females were postmenopausal age. It was found that 53.3% were euthyroid, 13.3% had hypothyroidism, 3.4% had hyperthyroid, 23.3% had subclinical hypothyroidism, and 6.7% had subclinical hyperthyroidism. Menopausal symptoms when compared to thyroid disorder demonstrated that somatic symptoms did not differ significantly across different thyroid disorder, psychological symptoms consisting of depressive mood was more prevalent among hypothyroid and hyperthyroid female (P<0.05), and urogenital symptoms consisting of sexual problems and bladder dysfunction did not differ significantly (P>0.05) in different thyroid disorder.

Conclusion

The present study concludes that thyroid disorder is common among pre- and postmenopausal age group women. Therefore, timely detection and intervention should be taken into consideration, and even if the thyroid level is found to be normal still lifestyle modification and counselling can help in improving the quality of life.

## Introduction

Perimenopause and menopause are natural phases of life for all women. It accounts for about 30% of India's female population [1]. A woman going through natural menopause will spend at least 30 years or more than 1/3 of her life in a hypoestrogenic state with long-term clinical and metabolic repercussions. Ninety percent of women report various symptoms in these years that include mental or physical slowing, fatigue, attention or concentration difficulties, forgetfulness, and mood disturbances. Low estrogen levels cause the majority of the symptoms that lead to poor life quality [1]. The symptoms of menopause can frequently be confused with those of hypothyroidism. It is common for hypothyroidism to go unrecognised or for its symptoms to be mistakenly attributed to menopause because both disorders are characterised by fatigue, mental or physical slowness, forgetfulness, difficulty concentrating, and mood problems. In contrast, hyperthyroidism is linked to significant problems like hot flashes, heat intolerance, palpitations, tachycardia, insomnia, and osteoporosis. It is important to note that thyroid dysfunction can have several clinical effects including depression, memory loss, cognitive impairment, and a variety of neuromuscular complaints. Even some women may experience subclinical hyperthyroidism or hypothyroidism [1].

Thyroid disorders are the second most prevalent disorders in the world after diabetes [2]. For appropriate reproductive physiology and behavior, thyroid hormone levels must be within normal ranges [2]. Thyroid-stimulating hormone (TSH) levels, particularly in females, may vary as a result of age-related changes in the body's regulatory mechanisms for thyroid hormone levels [3]. Additionally, there were few studies that showed the association of menopausal symptoms with thyroid disorders, while some studies did not demonstrate any correlation between menopausal symptoms with thyroid disorders [4-6]. Therefore, it is necessary to detect any abnormality in thyroid hormone levels in pre- and postmenopausal women so that early treatment can be initiated before the emergence of serious consequences. Hence, the aim of the present study was to determine the frequency of thyroid disorders in premenopausal and postmenopausal women and to find out its association with menopausal symptoms.

## Materials and methods

An analytical cross-sectional study was conducted after obtaining approval from the Institutional Ethical Committee (IEC) with IEC reference number 101/2021 from January 2021 to December 2022. All premenopausal and postmenopausal women attending the obstetrics and gynaecology department of a tertiary care center above 40 years of age, achieving natural menopause, and willing to participate were included in the study after receiving written informed consent. Whereas, women on hormone replacement therapy and that were known cases of thyroid disorder were excluded from the study. Based on the inclusion and exclusion criteria overall 150 participants were recruited for the present study. General and systemic examination was carried out, and the menopause rating scale (MRS) was used to evaluate for menopausal symptoms which is an 11-item survey having three dimensions that consist of the somatic domain, psychological domain, and urogenital domain [3]. The severity of menopausal symptoms was assessed according to the menopause symptom rating scale [3]. About 5ml of venous blood was collected in clean and dry, clot activator tubes that were sent to the central biochemistry laboratory of the institute to measure serum TSH, free T3, and free T4 levels. The sample was examined in the SIEMENS automated immunoassay machine, and a thyroid function test report was collected.

Data were entered in a Microsoft Excel sheet (Microsoft, Washington, USA) and were statistically analysed using SPSS Statistics version 24.0 for MS Windows (IBM Corp. Released 2016. IBM SPSS Statistics for Windows, Version 24.0. Armonk, NY: IBM Corp.) with epi info software version 7. Based on the analysis, the women were grouped into normal thyroid levels, hypothyroidism/hyperthyroidism, and subclinical hypothyroidism/hyperthyroidism to determine the frequency of thyroid disorders in pre- and post-menopausal women and their correlation with menopausal symptoms. The normal lab cutoff values consist of serum TSH range between 0.39 and 3.8Uiu/ml, Free T3 between 3.4 and 6.1pmol/L, and Free T4 between 9.8 and 18.8pmol/L. The data on continuous variables is provided as mean and standard deviation (SD), whereas the data on categorical variables were displayed as n (% of cases). The paired t-test and Mann-Whitney U test were applied. P ≤ 0.05 was considered as the significance level.

## Results

A total of 150 women were included in the study out of which 61 cases (40.7%) had premenopausal status and 89 cases (59.3%) had postmenopausal status in the study group. Out of the total participants, 71 cases (47.3%) were between the range of 40 and 50 years, 54 cases (36.0%) between 51 and 60 years, and 25 cases (16.7%) between 61 and 70 years in the study group. The mean ± SD of the age of cases studied was 51.93± 7.60 years, and the minimum-maximum age range was 40 - 70 years. The distribution of frequency of thyroid disorders among the cases studied is shown in Table 1. The distribution of frequency of thyroid disorder according to the menopausal status is demonstrated in Table 2.

**Table 1:** Distribution of frequency of thyroid disorders among the cases studied

Thyroid disorder	No. of cases	% of cases
Normal	80	53.3
Hypothyroidism	20	13.3
Hyperthyroidism	5	3.4
Sub-clinical hypothyroidism	35	23.3
Sub-clinical hyperthyroidism	10	6.7
Total	150	100.0

**Table 2:** Distribution of frequency of thyroid disorder according to menopausal status

Thyroid disorder	Premenopausal		Postmenopausal		Total		p-value
	n	%	n	%	n	%	0.737
Normal	49	55.5	31	50.0	80	53.3	
Hypothyroidism	10	11.2	10	16.7	20	13.3	
Hyperthyroidism	4	4.4	1	1.7	5	3.4	
Sub-clinical hypothyroidism	20	22.2	15	25.0	35	23.3	
Sub-clinical hyperthyroidism	6	6.7	4	6.6	10	6.7	
Total	89	100.0	61	100.0	150	100.0	

The distribution of association between thyroid disorder and somatic symptoms is demonstrated in Table 3, and the distribution of association between thyroid disorder and psychological symptoms is demonstrated in Table 4.

**Table 3:** Distribution of association between thyroid disorder and somatic symptoms NS: statistically non-significant

Thyroid disorder							
Somatic symptoms		Normal	Hypothyroidism	Hyperthyroidism	Sub-clinical hypothyroidism	Sub-clinical hyperthyroidism	p-value
		N (%)	N (%)	N (%)	N (%)	N (%)	
Hot flushes, sweating	Yes	4 (5.0)	2 (10)	2 (40)	2 (5.7)	1 (10)	0.063, NS
	No	76 (95.0)	18 (90)	3 (60)	33 (94.3)	9 (90)	
Heart discomfort	Yes	2 (2.5)	0 (0)	0 (0)	0 (0)	0 (0)	0.777, NS
	No	78 (97.5)	20 (100)	5 (100)	35 (100)	10 (100)	
Sleeping problem	Yes	5 (6.3)	3 (15)	1 (20)	0 (0)	0 (0)	0.112, NS
	No	75 (93.7)	17 (85)	4 (80)	35 (100)	10 (100)	
Joint and muscular discomfort	Yes	5 (6.3)	3 (15)	0 (0)	2 (5.7)	0 (0)	0.497, NS
	No	75 (93.7)	17 (85)	5 (100)	33 (94.3)	10 (100)	

**Table 4:** Distribution of association between thyroid disorder and psychological symptoms **p-value ≤ 0.05, NS: statistically non-significant

Thyroid disorder							
Psychological symptoms		Normal	Hypothyroidism	Hyperthyroidism	Sub-clinical hypothyroidism	Sub-clinical hyperthyroidism	p-value
		N (%)	N (%)	N (%)	N (%)	N (%)	
Depressive mood	Yes	2 (2.5)	4 (20)	1 (20)	0 (0)	0 (0)	0.003^**^
	No	78 (97.5)	16 (80)	4 (80)	35 (100)	10 (100)	
Irritability	Yes	5 (6.3)	0 (0)	1 (20)	0 (0)	0 (0)	0.131, NS
	No	75 (93.7)	20 (100)	4 (80)	35 (100)	10 (100)	
Anxiety	Yes	0 (0)	0 (0)	0 (0)	0 (0)	1 (10)	0.007^**^
	No	80 (100)	20 (100)	5 (100)	35 (100)	9 (90)	
Physical and mental exhaustion	Yes	2 (2.5)	5 (25)	0 (0)	2 (5.7)	0 (0)	0.004^**^
	No	78 (97.5)	15 (75)	5 (100)	33 (94.3)	10 (100)	

The distribution of association between thyroid disorder and urogenital symptoms is demonstrated in Table 5.

**Table 5:** Distribution of association between thyroid disorder and urogenital symptoms NS: statistically non-significant

Thyroid disorder							
Urogenital symptoms		Normal	Hypothyroidism	Hyperthyroidism	Sub-clinical hypothyroidism	Sub-clinical hyperthyroidism	p-value
		N (%)	N (%)	N (%)	N (%)	N (%)	
Sexual problems	Yes	2 (2.5)	2 (10)	0 (0)	1 (2.9)	0 (0)	0.482, NS
	No	78 (97.5)	18 (90)	5 (100)	34 (97.1)	10 (100)	
Bladder dysfunction	Yes	4 (5)	0 (0)	0 (0)	2 (5.7)	0 (0)	0.748, NS
	No	76 (95)	20 (100)	5 (100)	33 (94.3)	10 (100)	

## Discussion

The present study involved a total of 150 subjects out of which 61 were premenopausal and 89 were postmenopausal. It was found that 53.3% were normal cases, 13.3% had hypothyroidism which was also seen in many studies, 3.4% had hyperthyroidism, 23.3% had subclinical hypothyroidism, and 6.7% had subclinical hyperthyroidism. The present study also compared each of these different thyroid disorders with different menopausal symptoms. In order to find out if there is any association between thyroid disorder and menopausal symptoms, the menopausal symptoms were sub-classified into three groups consisting of somatic symptoms, psychological symptoms, and urogenital symptoms. Out of these, somatic symptoms did not differ significantly with thyroid functioning status along with the euthyroid group. In psychological symptoms, the depressive mood was more prevalent among hypothyroid and hyperthyroid females, whereas it was not so prevalent in the euthyroid, subclinical hypothyroid, and subclinical hyperthyroid groups. Anxiety was seen only in one female with the hyperthyroid disorder in comparison to other disorders, and physical and mental exhaustion was also more in hypothyroid females. Urogenital symptoms including sexual problems and bladder dysfunction did not demonstrate statistically significant results.

Several studies reported menopausal symptoms and their correlation with thyroid disorder, but the data was either insufficient or cannot be compared with one another. This is because different authors have used different definitions of the various clinical conditions, the criteria used to choose patients, the effects of age and sex, the coexistence of environmental factors, and many other forms of thyroid disease [7-9]. The interpretation of the functional outcome of the thyroid should be made with caution during the premenopausal and postmenopausal periods [10-12]. Some studies only tested thyroid function disorder and did not correlate it with menopausal symptoms and some only tested women who are postmenopausal, whereas other studies compared pre- and postmenopausal results [13-16]. In addition, research shows that the prevalence of thyroid disorders is more with advancing age [13-15]. As a direct consequence of this, women are more likely to suffer from thyroid disorders [13-16].

A previous study given by Aryal et al. concluded that patients above the age of 70 who have TSH levels below 10mIU/l should undergo TFT and surveillance every six months as 25% of the 825 individuals recruited had thyroid problems which were more prevalent in women than in men [17]. Hypothyroidism (8%) and subclinical hypothyroidism (8%) had a higher prevalence than subclinical hyperthyroidism (6%) and hyperthyroidism (3%). Additionally, those older than 30 were more likely to experience thyroid-related issues [17]. The results of this study, which are in line with past results in other populations, revealed a higher prevalence of abnormal thyroid function. The most prevalent condition is subclinical hyperthyroidism, which is followed by subclinical hypothyroidism and hypothyroidism. Women and older persons are more likely than the general population to experience thyroid issues [17].

Additionally, a study given by Shaikh et al. reported that nearly one in eight women may experience a thyroid condition at some point in their lives. The risk of discovering subclinical hypothyroidism rises with age; the typical age of a person with hypothyroidism was found to be 58 years old. Clinically, it can be difficult to distinguish between postmenopausal symptoms and thyroid disease symptoms. There are currently no reliable recommendations for evaluating menopausal women for thyroid function. In this study, hypothyroidism prevalence among postmenopausal women was estimated. In total, 100 patients were included in this investigation and discovered that 17% of the female patients who were grouped by age and BMI had hypothyroidism. Age at menopause, BMI, TSH, T3 and T4, weariness, muscle cramps, depression, weight gain, cold sensitivity, and sleeping issues are all associated with hypothyroidism [18]. Chandrashekhar found that increasing age and postmenopausal status are at increased risk for thyroid dysfunction. Hence, this indicates the need to check for thyroid function in patients with symptoms of menopause [2].

Bordoloi et al. conducted a study in which serum TSH levels were tested in 304 females. If the TSH level was abnormal, unbound T3 and T4 levels were tested. TSH levels were examined between different age groups, and it was discovered that 8.2% of premenopausal and 12.7% of postmenopausal women had hypothyroidism. Subclinical hypothyroidism was more common than clinical hypothyroidism and postmenopausal females have decreased thyroid function [3]. A study given by Kolanu et al. concluded that the older post-menopausal women’s mean serum TSH levels (3.39+2.45) was discovered to be greater than those reported in pre-menopausal women (2.60+1.31). The results demonstrated that older post-menopausal women have higher TSH activity [15].

Shetty et al. determined the prevalence and incidence of thyroid disorders, which were found to be influenced by sex and age and to be more prevalent in women and older adults. The study concluded that postmenopausal women should have their serum T3, T4, and TSH levels checked to prevent thyroid dysfunction. Thus, an association between thyroid disorders and postmenopausal women was discovered; however, menopausal symptoms were not examined in their study [19]. The limitations of the study mainly involved a small sample size which can be overcome in future studies. Whereas, the correlation between age and TSH levels and the correlation between MRS and TSH levels can be considered for the future scope of the study. Furthermore, the study suggests checking thyroid levels when patients are exhibiting symptoms of menopause.

## Conclusions

The present study concludes that thyroid disorder is common among pre- and postmenopausal age group women. Subclinical hypothyroidism was most common followed by hypothyroidism, subclinical hyperthyroidism, and hyperthyroidism. Additionally, psychological symptoms consisting of depressive mood were more prevalent in women with a thyroid disorder. Hence, the symptoms of menopause can frequently be confused with those of hypothyroidism which can go unrecognised and can be considered as menopause. Therefore, routine checking of thyroid levels should be taken into consideration for early intervention, and even if thyroid levels are found to be normal still lifestyle modification and counseling can help in improving the quality of life.
